# Nano titania aided clustering and adhesion of beneficial bacteria to plant roots to enhance crop growth and stress management

**DOI:** 10.1038/srep10146

**Published:** 2015-05-13

**Authors:** N. G. M. Palmqvist, S. Bejai, J. Meijer, G. A. Seisenbaeva, V. G. Kessler

**Affiliations:** 1 Department of Chemistry and Biotechnology, Swedish University of Agriculture, BioCenter SLU, Box 7015, 75007 Uppsala, Sweden; 2 Department of Plant Biology, Swedish University of Agriculture, BioCenter and Linnean center of Plant Biology SLU, Box 7080, 75007 Uppsala, Sweden; 3 Captigel AB, Virdings allé 32b, 754 50 Uppsala, Sweden

## Abstract

A novel use of Titania nanoparticles as agents in the nano interface interaction between a beneficial plant growth promoting bacterium (*Bacillus amyloliquefaciens* UCMB5113) and oilseed rape plants (*Brassica napus*) for protection against the fungal pathogen *Alternaria brassicae* is presented. Two different TiO_2_ nanoparticle material were produced by the Sol-Gel approach, one using the patented Captigel method and the other one applying TiBALDH precursor. The particles were characterized by transmission electron microscopy, thermogravimetric analysis, X-ray diffraction, dynamic light scattering and nano particle tracking analysis. Scanning electron microscopy showed that the bacterium was living in clusters on the roots and the combined energy-dispersive X-ray spectroscopy analysis revealed that titanium was present in these cluster formations. Confocal laser scanning microscopy further demonstrated an increased bacterial colonization of *Arabidopsis thaliana* roots and a semi-quantitative microscopic assay confirmed an increased bacterial adhesion to the roots. An increased amount of adhered bacteria was further confirmed by quantitative fluorescence measurements. The degree of infection by the fungus was measured and quantified by real-time-qPCR. Results showed that Titania nanoparticles increased adhesion of beneficial bacteria on to the roots of oilseed rape and protected the plants against infection.

Titania (TiO_2_) nanoparticles (TNs) have become one of the most important substances in nanotechnology. Many studies have been conducted into the properties of various TNs in solutions to estimate their behaviour. The effect of size, pH, ionic strength, crystal phase and presence of proteins and other biomolecules on agglomeration, aggregation, hydrodynamic size and the behaviour of nanoparticles in solution have been thoroughly investigated^1^,^2^,^3^,^4^,^5^,^6^,^7^. Lately interests and ideas have emerged to apply TNs in agricultural practices from pesticide formulations to growth stimulators^8^,^9^,^10^,^11^. There is a pronounced interest into the effect of TNs as reagents in various environments^12^,^13^,^14^,^15^. Titania nanoparticles have been demonstrated to have adhesive effects on bacteria. *Chowdhury I. et al. (2012)*^16^ showed that *Escherichia coli* and humic acids in effluent aqueous systems have significant effects on aggregation and deposition of TNs. *Webster et al. (2008)*^17^ found that nano structured titania increased adhesion of bacteria on to surfaces compared to unstructured bulk titania. It can be hypothesised that these properties of TNs could be used to guide bacteria to a place where they are wanted. Titania nanoparticles are considered to be safe and are even used with up to 30% concentration in skin products^18^. Titania nanoparticles are also considered safe for human consumption and are used in EU as a food colorant E171.

There have been reported several approaches for preparation of stable titania colloids for use in biological applications. Ammonium oxo-lactato-titanate, (NH_4_)_8_Ti_4_O_4_(Lactate)_8_·4H_2_O (commercial name TiBALDH, an abbreviation of the erroneous interpretation of the structure as Titanium-Bis-Ammonium-Lactato-Dihydrohyde) is known to produce via a solution equilibrium reaction monodispersed crystalline TNs (spherical, about 3 nm), which are highly water soluble due to surface capping with lactate ligands. It has been proposed that this equilibrium can occur on interaction of the plant root exudates with soil minerals^19^. CaptiGel technology refers to production of stable aqueous hydrosols and hydrogels of metal oxides, in particular, titania, via acidic hydrolysis of metal alkoxides modified with hydrophilic and electrically chargeable ligands such as triethanolamine. The size of the spherical TNs obtained by this approach is about 3.5 nm thus resembling the nanoparticles obtained from TiBALDH. These both types of nano particles have been proven to be biocompatible with lung cell cultures and pollen grains^20^. Titania nanoparticles seem environmentally benign and convey properties that might make them useful as protective encapsulating agents that increase adhesive forces in the interspecies relationship between bacteria and plants.

*Bacillus amyloliquefaciens* is a well-studied rhizosphere species where several strains have had their genome sequenced^21^,^22^,^23^. Certain strains serve as plant growth promoting rhizobacteria (PGPR) and improve stress management of plants serving as biocontrol agents. The UCMB 5113 strain has shown to be a very promising candidate for plant protection^24^,^25^. It suppressed disease caused by several fungal pathogens in *Brassica napus*^24^ and could also reduce abiotic stress such as drought in wheat (*Triticum aestivum)*^26^. While rhizobacteria are promising bio control agents for future applications in plant protection their efficacy is variable in field conditions. Hence bacterial formulations that can protect, aid and augment bio control will be of prodigious benefit to agriculture.

Surface charges of both bacteria and nanoparticles vary. Titania nanoparticles have a zeta potential that goes from positive to negative as pH increases with zero potential around pH 6 according to *Jiang J. et al. 2008*^2^. Bacteria are carrying both negative and positive charges with a general dominance towards total negative net charge^27^. The attachment of bacteria to a surface is rather implausible. If one ignores flagella and chemo-taxis, the Brownian motion might be a stronger physical force than the attractive van der Waals, electric, or hydrophobic forces between the bacteria and another surface^28^. It has been shown that positively charged nanoparticles can play a role as glue between bacteria^29^. Roots surface charge varies between species but according to *Kinraide T.B. and Wang P. (2010)*^30^ a mean value can be estimated around -30 mC m^−1^.

There are many examples of both plants and microorganisms that produce nanomaterials in nature^31^,^32^,^33^,^34^,^35^. It is well-known that these naturally occurring metal-oxide nanomaterials possess enhanced affinity to phosphate and phosphonate ligands, which may act as a mechanism for enhancing aggregation of single cell organisms and function as glue between bacteria and roots^36^,^37^. It has recently been demonstrated that the TNs can emerge from solution equilibrium relevant for the conditions around the roots of (especially juvenile) plants.

Here we present evidence supported by the data from a variety of qualitative and quantitative methods that negatively charged TNs synthesised by Sol-Gel synthesis convey the novel use as facilitator of the colonization of oilseed rape roots by the PGPR and bio control agent *B. amyloliquefaciens* UCMB5113.

## Results

### Characteristics of nanoparticles

**Figure 1:**
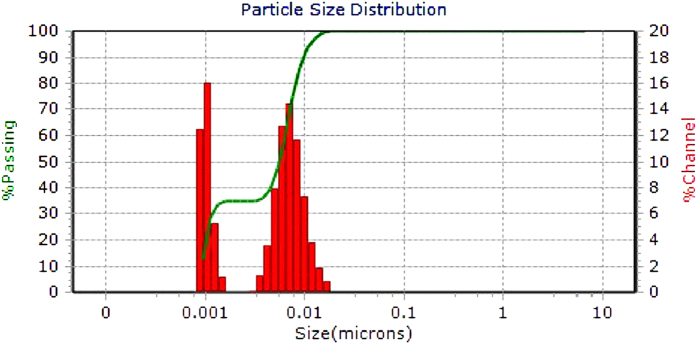
Particle size distribution of TiBALDH-derived nanoparticles in M9-solution as seen with DLS.

Both types of nanoparticles were found to exist as aggregates five to ten nanometers in size according to TEM (Supplementary Figure S1). The nanoparticle tracking analysis (NTA) showed a mean particle size of 208 nm in water solution and 238 nm in M9 minimal salts solution. However the NTA cannot detect particles smaller than 10-20 nm, especially if there are simultaneously larger particles present with greater ability to scatter light (Supplementary Figure S2). The NANO-flex instrument using dynamic light scattering can distinguish the smaller particles and indeed we could see a peak around 7 nm and 300 nm in the DLS analysis (Fig. 1). The Captigel particles and the TiBALDH particles measured, 8.37 mV and −9.82 mV in milli-Q water and −17.55 mV and −22.2 mV in M9 minimal salts solution, zeta potential respectively. X-ray diffraction showed that the TiBALDH synthesized particles were of anatase crystal phase and the Captigel particles showed a much weaker crystallinity mostly resembling anatase (Supplementary Figure S3).

### Bacterial growth in presence of nanoparticles

**Figure 2:**
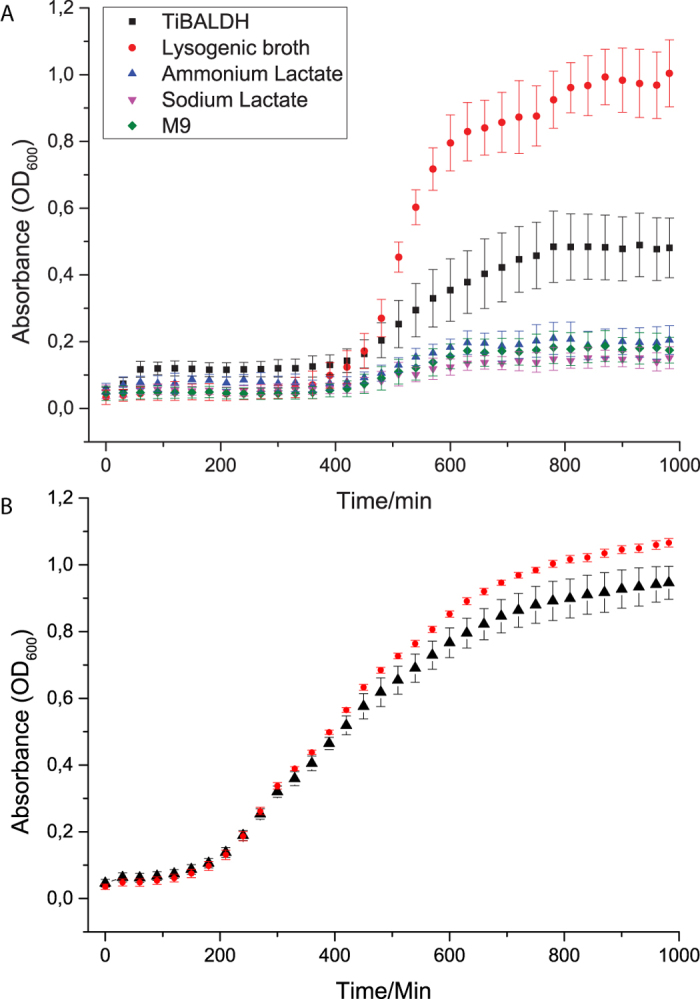
Growth of bacteria as measured by absorbance (OD_600_) over time in minutes. Values represent mean values of eight biological replicates ± SD. **Panel A.** TiBALDH derived nanoparticles. Bacteria grown in M9 medium together with 50 μg TNs is noted with a square ■. Positive control with sodium lactate is noted with an upside down triangle ▲, ammonium lactate is noted with a triangle ▲, and bacteria grown in LB-medium noted by a full circle • and negative control with the bacterium grown in M9 is noted by a diamond ♦. **Panel B.** Captigel derived nanoparticles.

Absorbance measurements showed a clear increase in absorbance from bacterial cultures where TiBALDH nanoparticles were added. Nanoparticles alone did not absorb any light at λ 600 nm (results not shown). The positive controls with lactate were not different from the negative control, showing that it was not an effect of added nutrition, in the form of lactate, giving increased absorbance on addition of TiBALDH derived TNs. The Captigel TNs also increased absorbance of bacterial cultures in LB-medium (Fig. 2).

The cultures collected after growth with TiBALDH TNS in the plate reader and serially diluted on LB plates could not be quantified with this method. The TNs adhered the bacteria to each other so that single colony forming units were mired in clusters and grew as few big patches (Supplementary Figure S4). The colonies were held together by the TNs so strongly that it was difficult, but not impossible, to open up these clusters mechanically for imaging. The SEM images of the cultures showed bacteria growing inside these dense clusters and EDS-analysis confirmed that TNs were an integral part of the same clusters (Supplementary Figure S5 and supplementary Vid. SV1).

Since there was a clear difference in growth curves as measured by absorbance a protein investigation was conducted with TiBALDH TNs. A sodium dodecyle sulfate – polyacrylamide gel electrophoresis (SDS-PAGE) indicated a difference in amount of protein, between bacteria grown together with TNs and bacteria only grown in M9 medium (Supplementary Figure S6). However, a subsequent 2D-gel electrophoresis study did not show any differences in protein profile between bacteria grown with or without TNs (Supplementary Figure S7).

### Bacterial root colonization

**Figure 3:**
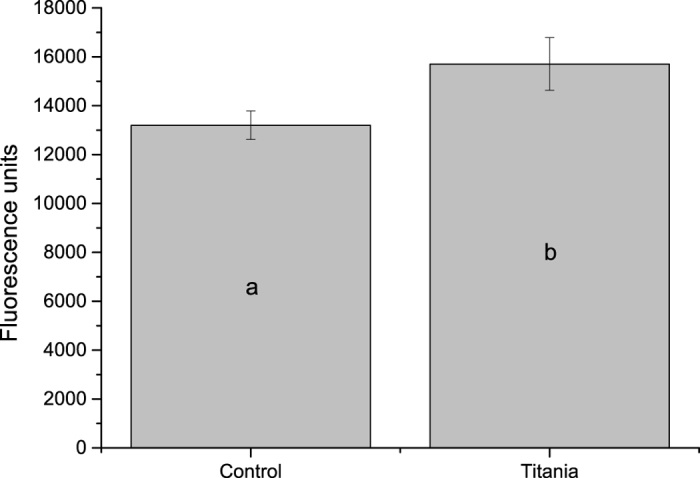
Amount of bacteria washed off of roots treated with TiBALDH TNs as measured by fluorescence intensity. A statistically significant difference was found indicated by different letters (P-value 0.040 n = 8).

**Figure 4:**
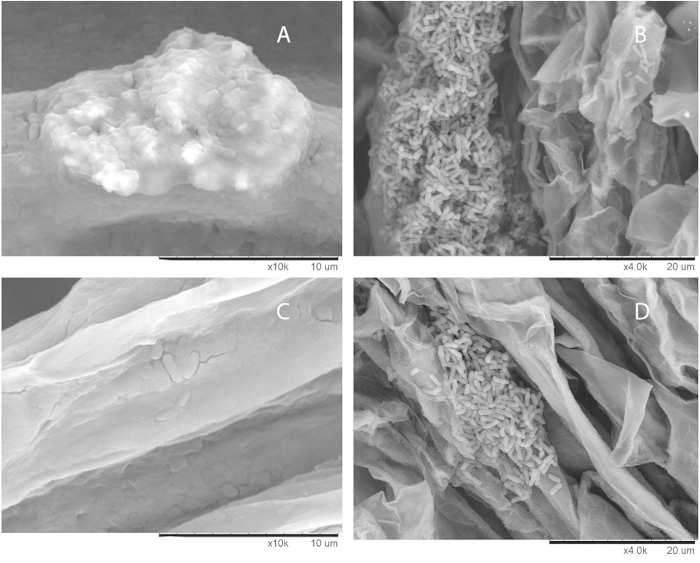
SEM-images of *B. amyloliquefaciens* on oilseed rape roots. **A** Bacteria together with Captigel nanoparticles (See supplementary figure 5 for EDS-data). **B** Bacteria together with TiBALDH nanoparticles (See supplementary figure 5 for EDS-data). **C** Control for image A with bacteria found in the same region of the root, namely the base, of plant treated with bacteria only. **D** Control for image B with bacteria found in the same region of the root, namely the base, of a plant treated with bacillus only.

**Figure 5:**
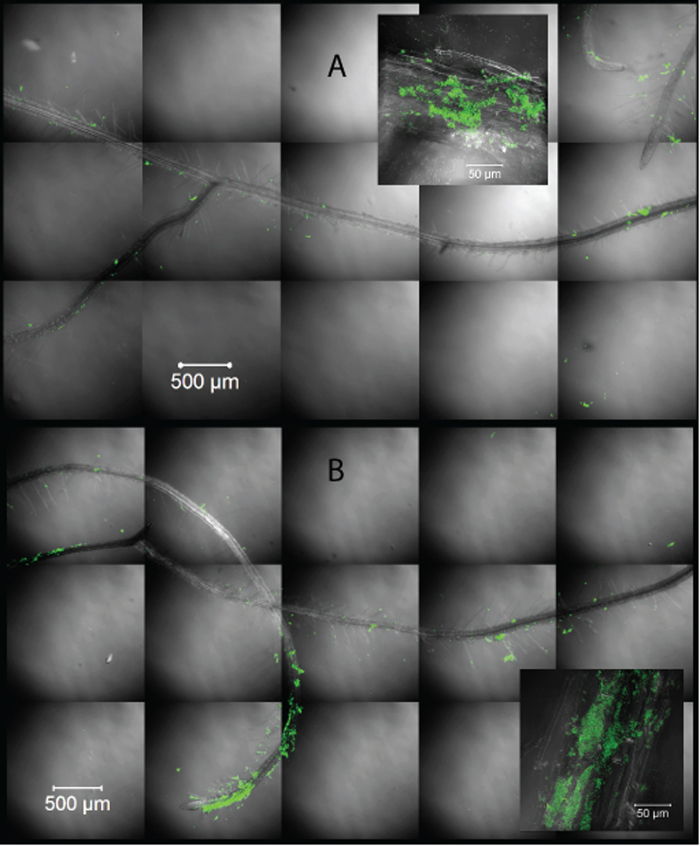
Efficiency of adhesion of bacteria to roots in absence (**A**) or presence (**B**) of TiBALDH TNs. Confocal laser scanning images composed out of four z-layers to a depth of 12 μm. The inset with 630 times magnification was taken on a random spot of the bigger image.

Amount of bacteria according to fluorescence measurements had a statistically significant increase for bacteria recuperated from roots treated with TiBALDH TNs (Fig. 3). We also studied plant roots that had been dipped in solution with TNs and *B. amyloliquefaciens* by both SEM and CLSM. The SEM-images show lumps of bacteria present on the root in clusters (Fig. 4) with measurable titanium content according to the EDS-analysis (Supplementary Figure S8). In the CLSM-images a clear increase of the number of bacteria surrounding the root is shown (Fig. 5). A higher degree of clustering of bacteria and a higher degree of adhesion when TiBALDH TNs are present can be seen in the image.

The quantification of bacteria colonizing plant roots with colony counting and semi-quantitative SEM-method indicated that an increased adhesion of bacteria occurred when TNs were introduced to the system. The colony forming unit per mg of root was higher for the control but the amount of bacteria left on the roots after washing was greater with TiBALDH TNs and a statistically significant increase in the amount of colonies left on the roots was found (Supplementary Tab. ST2).

### Pathogen bio assay

**Figure 6:**
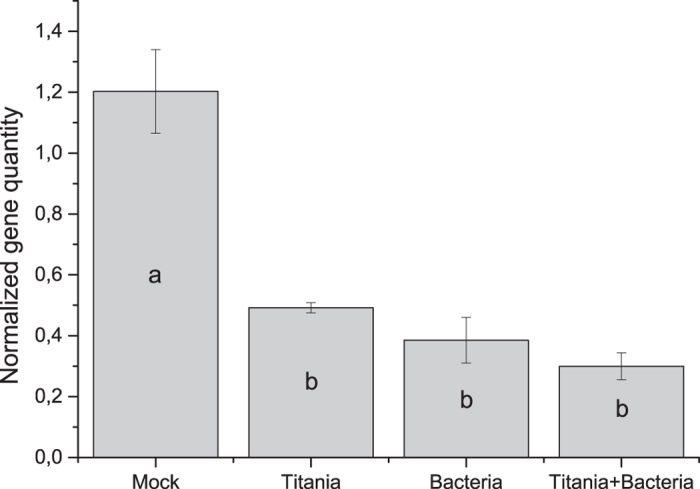
*A. brassicicola* gene quantity normalized towards plant gene Ubiquitin 5 extracted from leaves treated with bacteria and TiBALDH TNs. Different letters shows statistically significant difference (P-value < 0.000 n = 3).

**Figure 7:**
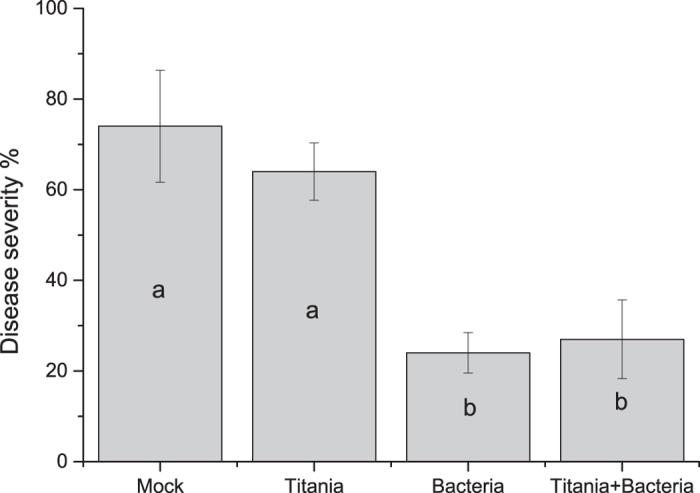
Disease severity score made with visual estimation of the plants. Treatments are shown as follows: Mock control on the left, positive control with TiBALDH TNs second from the left, bacteria only third from the left and finally bacteria with TiBALDH TNs furthest to the right. Different letters shows statistically significant difference (P-value < 0.000 n = 3).

In order to investigate whether the TN- treated Bacillus had any effect on plant protection, we challenge-inoculated leaves of the treated and non-treated plants with the necrotrophic pathogen *Alternaria brassicicola.* The quantification of the amount of pathogene DNA by qPCR technique showed a statistically significant difference between negative control and all the other treatments. This means that the amount of fungal organisms was decreased in the leaves as the result of treatment with either TiBALDH TNs, biocontrol bacteria or both (Fig. 6). There was no statistically significant difference between bacteria only and the combination with TNs although the average was lower when the bacteria had been mixed with nanoparticles. The disease scoring showed no phenotypical difference between mock and positive control with TNs but there was a statistically significant difference between treatments with bacteria and without bacteria (Fig. 7).

## Discussion

**Figure 8:**
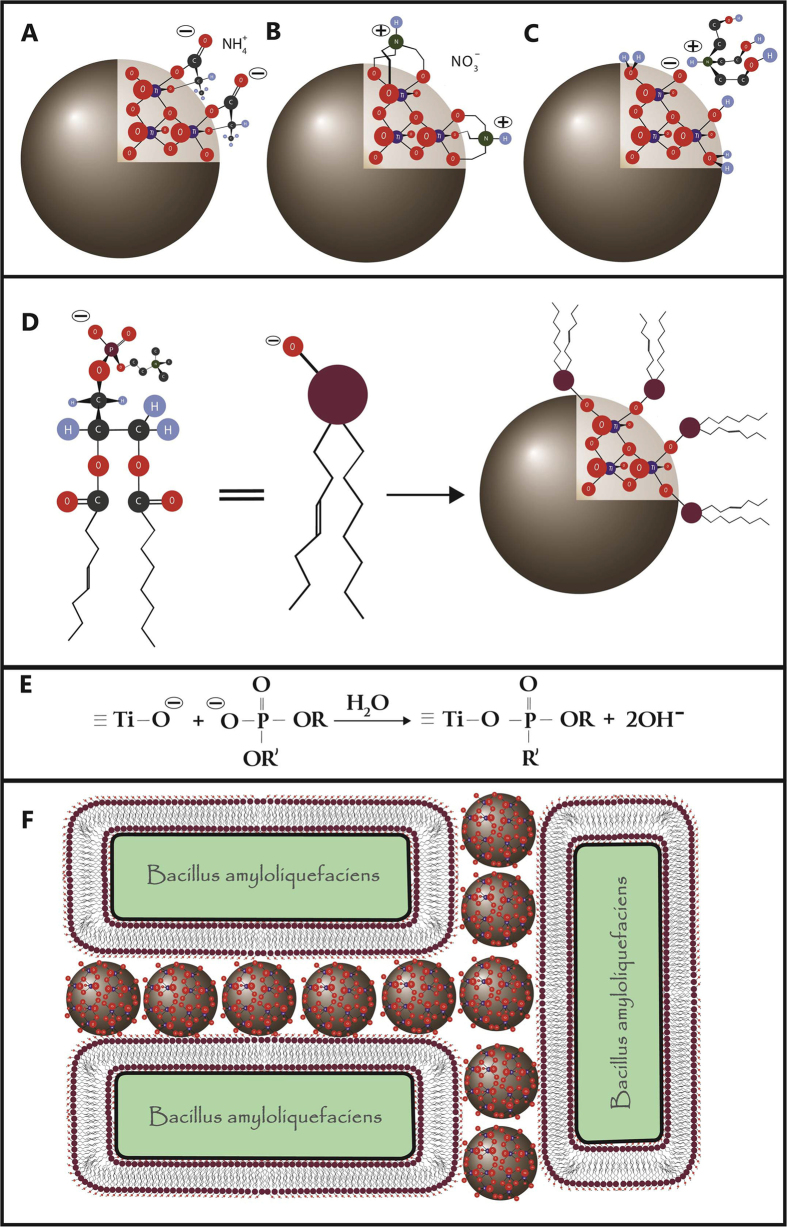
Schematic presentation of the TiBALDH derived particles (**A**) CaptiGel particles in organic medium (**B**) CaptiGel particles in water (**C**) formation of the inner sphere complexes of TNs with phospholipids (**D**) equation for the surface reaction between TNs and phospholipids (**E**) and cluster formation for bacteria initiated by TNs (**F**).

We have applied in our essays in parallel two types of titania nanoparticles generated by molecular level processes with almost identical size, 3 nm for those derived from TiBALDH and ca. 3.5 nm in CaptiGel. The surface area, often considered to be a key factor in interactions with nanomaterials, estimated by geometric calculation assuming the observed spherical geometry, constitutes 520 m^2^/g and 450 m^2^/g for TiBALDH and CaptiGel particles respectively and is of the same order of magnitude. Unfortunately, experimental determination of the exact surface area for not fully crystalline colloids is essentially impossible in the view of their aggregation and transformation on drying (required for measurements). An important feature shown by both colloids is that they display both in water and in the applied medium negative zeta-potentials of comparable value. The stabilization of the negative surface charge is achieved in apparently different ways for the two colloids. The surface of TiBALDH derived particles is covered by lactate ions involved into inner-sphere complexes, according to the earlier NMR studies(see, Fig. 8A)^19^ The CaptiGel particles have been described structurally, using molecular model compounds, in organic medium, where they are positively charged due to protonation of a part of chelating triethanolamine ligands on the surface (Fig. 8B)^38^. Dissolution in water leads to apparent loss of a part of the organic layer on CaptiGel particles (according to TGA) and their re-charging. It has to be concluded that the triethanolammonium salts, responsible for the positive charge in organic medium, are “washed” away. Electrospray MS analysis of aqueous CaptiGel shows the presence of triethanolammonium ions, indicating that the particles have them in the outer sphere complex (Fig. 8C). Interaction of the particles with cell membranes occurs apparently not via simple electrostatic interactions as both surfaces are charged negatively. A plausible way of the binding which has been proven efficient to both plant roots and to gram-positive microorganisms, should be related to the surface chemistry of these biological objects. Their clear common feature is that they have phospholipid membrane fragments exposed. It is reasonable to suppose that the interaction of both types of titania with the latter should occur via formation of inner-sphere complexes (see Fig. 8D,E) with the phosphate function in the membrane^37^. Comparable size and analogous interaction mechanisms make both types of particles to provide closely analogous effects (see Fig. 8F). We were not able to distinguish the action of the two types of particles in morphology or quantitative effects, which in continuation are denoted by a common term (small) titania nanoparticles (TNs).

Our aim was to investigate if TNs could be used to support beneficial rhizobacteria in the colonization of plant roots. There was a clear and substantial increase of 600 nm light absorbance when the *B. amyloliquefaciens* 5113 was cultured together with TNs. These high absorbance values could not be seen with TNs alone or bacteria alone. This means that there is an interaction, between nanoparticles and bacteria, which strongly increases absorbance. One can hypothesize that there is an increase of clustering that the bacteria cannot achieve by themselves or that there is increased growth. Indeed it was possible to see bacteria living and dividing in a network together with the particles by SEM after growth on agar plates. Analysis by SEM and EDS of *Bacillus* 5113 on roots revealed that the clusters formed on the roots also contained titanium. Not only did the nanoparticles aid in clustering of the bacteria but CLSM showed that the colonization of the roots also was enhanced. The roots were covered to a higher extent with bacteria when the bacteria had been treated with TNs. It is visible in the images that the colonies are appreciably bigger and maybe even more highly ordered. The SDS-PAGE results with thicker protein bands on TN-treatment further suggests an increased growth of bacteria together with the nanoparticles. It also shows that there is no stress response induced on the bacteria since no difference in protein profile was observed compared to control which was further confirmed by 2D-gel electrophoresis. The quantification by viable count did not lead to any statistically significant difference in results. However, together with the semi-quantitative assay interesting results were found that comply with our hypothesis, being also in agreement with the results of *Larsen M.U et al.* 2009^29^. We suggest that first the nanoparticles helped bacteria colonize the plants creating greater biomass of bacteria. However the amount of colony forming units washed off per root mg was less because a statistically significant increase in amount of bacteria was still strongly adhered to the roots by the TNs. The fluorescence measurements did show a statistically significant increase in amount of bacteria washed off from roots despite this strong adhesive effect instigated by the TNs. The fluorescence results are related to the absolute numbers of bacteria and are not affected by their clustering. The growth of fungi in the TN-treated leaves of oilseed rape seems to be affected by the plant root treatment by TNs in a complex way. The data indicated that the TNs could cause reduction of the fungal growth inside the leaves but that its virulence effectors were still present and caused similar disease development as compared to control. When the bacteria are present both the growth and the virulence of the fungi is suppressed.

## Conclusions

The obtained results provide a preliminary proof-of-concept for the idea that the Sol-Gel synthesized titania nanoparticles in certain concentrations might be utilized to improve bacterial formulations. The TNs evidently have an effect on the growth and behavior of *Bacillus amyloliquefaciens*. The presence of TNs did increase colonization of roots in liquid medium. The nanoparticles seem to aid the bacterium to adhere to plant roots, possibly increasing survival and fitness as competitor for space and resources in the rhizosphere of agricultural plants. This can be of great aid for formulations of bio-control agents improving their efficiency against fungal infections. It remains, however, to be proven if the increased colonization of the roots also can occur in soil. The difference between TiBALDH and CaptiGel derived nanoparticles was not possible to distinguish, but it is important to keep in mind that CaptiGel consists entirely of TNs^38^, while they have to be extracted from TiBALDH (and the maximal yield is less than 10 wt %)^19^.

## Method section

### Synthesis and characterization of nanoparticles

Titania nanoparticles were synthesized from TiBALDH (purchased from Sigma Aldrich, CAS No. 65104-06-5) through a simple precipitation method described in *Seisenbaeva G. et al.* 2013^19^ and from titanium tetraethoxide (CAS No. 3087-36-3) by the techniques of the Captigel patent no. WO07145573. The particles were washed by centrifugation at 4000 rpm three times in anhydrous ethanol and then washed in water four times. They were analysed by Thermo Gravimetric Analysis (TGA) in a Perkin-Elmer Pyris 1, X-Ray Powder Diffraction in Bruker SMART Apex-II multipurpose diffractometer operating with MoKα radiation λ = 0.71073, Energy-Dispersive X-ray Spectroscopy (EDS) and scanning electron microscopy (SEM) with an Hitachi TM-1000-μDeX. Transmission electron microscopy was used to determine the size of the particles using a Philips CM-20 Super Twin microscope, operating at 200 kV. The particle size in solution, both deionized water and M9-minimal salts, was investigated with nano tracking analysis with Nanosight 300 and additional investigation into the different aggregation-states of the particles analysed by dynamic light scattering (DLS) using Microtrac NANO-flex. The zeta potentials were measured on a Malvern Nano ZS.

### Selection of standard treatments

After growing bacteria together with TNs and assessing growth through standard optical density (OD_600_) absorbance measurements and exposing seeds to different concentrations of the titania nanoparticles and assessing the germination rate we concluded that 50 μg TN ml^−1^ would be the optimal concentration for our purposes (Supplementary Figure S10). Four standard treatments were constructed; A: Mock with deionised and autoclaved water, B: positive control with 50 μg  ml^−1^ of titania nanoparticles, C: positive control with only the bacterium adjusted to 10^7^ colony forming units (CFU) ml^−1^ and D: the treatment with both nanoparticles and bacterium.

### Bacterial growth in presence of nanoparticles

*B. amyloliquefaciens* subsp. *plantarum* UCMB5113 (referred to as 5113) was grown in standard M9 minimal salts medium with titania nanoparticles in a round bottom 96 well plate with shaking at 28 °C for 16 hours in a FLUOstar Omega plate reader. Since the TiBALDH nanoparticles are surface capped by lactate ligands, ammonium lactate and sodium lactate only was added as controls. The concentrations were aligned with the concentrations of lactate on the TiBALDH nanoparticles in solution and calculated after the results from thermo gravimetric analysis (results not shown).

### Growth and bacterial treatment of plants

The seeds of Oilseed rape cultivar Westar were sterilized in 70% ethanol for one minute and 10% chlorine for three minutes and later washed in autoclaved deionized water for five times three minutes. Seeds were spread on Murashige and Skoog agar medium plates for germination and kept in growth chamber at 25 °C with twelve hours light. Seven to fourteen days after germination, ten replicate seedlings were selected with uniformity in mind. The seedling roots were left to soak for one hour in petri dishes, with the four standard solutions, before being transferred to plates with Murashige and Skoog medium or to pots with sterilized soil for growth in chamber at 25 °C with twelve hours light with 200 μmol m^2^ s^−1^ intensity. The plants that were analyzed with SEM grew for seven days after treatment in plates. Plants used for bio assay were grown in sterilized soil for 14 days after treatment before they were inoculated with the pathogen and harvested seven days later. Plant roots that were imaged with confocal laser scanning microscopy (CLSM) were left to soak for four hours in the treatments before being brought directly to the microscope for investigation.

### Semi-Quantification of bacteria on roots

To quantify bacteria twelve replicates with four seedlings each from a nine by nine cm square plates were subjected to standard treatment C and D. After one week the roots were washed by keeping the roots in 0.1 M phosphate buffer for one hour on a shaker and finally by vortexing for 20 seconds. The roots were then transferred to 0.1 M citrate buffer and the same procedure was repeated. The roots were then left to air dry in Petri dishes and buffer solutions were mixed and centrifuged to pellet the bacteria and resuspend them in water. The bacterial solutions were serially diluted and spread on LB-agar plates and CFUs were counted. A semi-quantitative method with SEM to compare the amount of bacteria left on the roots was conducted, 30 random images of each replicate were taken and presence or non-presence of bacteria or bacterial colonies was noted.

### Bacterial plant root colonization – SEM-imaging

The roots were removed from the agar plates and separated from the leaves. The roots were fixed in 2% glutaraldehyde in 0.075 M phosphate buffer as described by *Yuan J. et al.* 2013^39^. Bacterial cultures from the plate reader were imaged after 48 hours of growth on LB-plates, after being sputtered with gold. The roots that were air dried after colony counts were sputtered with gold before imaging. All editing of images, brightness and contrast enhancement only, was made with Photoshop and Illustrator CS6.

### Bacterial plant root colonization – Confocal fluorescence imaging

Visualization of the root colonization by the bacteria were made by enhanced green fluorescence protein (EGFP) tagging the bacterium and imaging in an inverted Zeiss LSM 780 with 10x plan-Apochromat objective with numerical aperture 0.45 and 63x C-Apochromat water immersion with numerical aperture 1.2. Pinhole was set to 1 AU and an argon ion laser was run at 488 nm with 2.0% power. All image processing was done equally for all images with Zeiss ZenBlack software.

### Bacterial plant root colonization – Fluorescence measurements

First linear regression curves with dilution series of EGFP-transformed bacteria in water, in citrate buffer and in solution with titania nanoparticles were constructed. The r^2^-values of the curves were over 0.98 and no difference between the solutions was found. Plants were grown in plant culture magenta jars with a metal mesh and liquid Murashige and Skoog medium. After five days the Murashige and Skoog medium was poured out and replaced with milli-Q water with the bacterium set to OD_600_ 0.4 with or without titania nanoparticles at 50 μg/ml concentration. After four hours the roots were collected and six plants were pooled into each out of eight replicate. The roots were put into 50 ml falcon tubes with 0.5 M pH 6.0 citrate buffer, briefly vortexed and left on a shaker at 450 rpm for one hour. The roots were collected and dried at 37 °C to be weighed. The falcon tubes were centrifuged and the pellet was re-suspended in milli-Q water centrifuged again and then re-suspended in 1 ml milli-Q water. From this 250 μl of each replicate was transferred into 96-well plate and measured for fluorescence in a FLUOstar Omega plate reader.

### Pathogen bioassay

Seedlings were after treatment with *Bacillus* and TNs transferred into 10*10 cm pots with sterilized soil. *Alternaria brassicicola* strain MUCL20297 was grown on potato dextrose agar plates for 2 weeks at 22 °C. Spores were collected and suspended in water (5 × 10^5^ spores ml^−1^). *A. brassicicola* inoculation was performed by adding 5 μl drops onto the leaf surface as previously described by *Thomma et al.,* 1998^40^. Two leaves per plant were inoculated with two 1 μl drops each. Three plants were pooled and taken as one biological replicate and three biological replicates were used. Five days after challenge, disease severity was scored and samples were collected for pathogen quantification. Disease rating was assessed on the basis of symptom severity as described by *Van der Ent et al.* 2008^41^. The infection areas were cut out in equal squares and snap freezed for DNA extraction using DNeasy plant mini kit. Sufficient DNA concentration was verified with Nanodrop absorbance measurements. *A. brassicicola* DNA quantification was carried out by qPCR of a cutinase gene (ABU03393) and normalized against *A. thaliana* ubiquitin 5 gene as reference DNA (Supplementary table S2).

### Statistics

All statistics were performed using Minitab 16. An Anderson darling test confirmed normal distribution of the data and test for equal variances confirmed that variances were equal. A generalized linear model was run with all treatments, using the Tukey method to compare means.

## Additional Information

**How to cite this article**: Palmqvist, N. G. M. *et al*. Nano titania aided clustering and adhesion of beneficial bacteria to plant roots to enhance crop growth and stress management. *Sci. Rep.* **5**, 10146; doi: 10.1038/srep10146 (2015).

## Supplementary Material

Supplementary Information

Supplementary Video S1
