# Supplementary material for: Nano titania aided clustering and adhesion of beneficial bacteria to plant roots to enhance crop growth and stress management
Source: Sci Rep. 2015 May 13;5:10146. doi: 10.1038/srep10146 (PMC4650812; doi:10.1038/srep10146)
Supplement: Supplementary Information [file srep10146-s1.pdf]

Supplementary data

Title: Nano titania aided clustering and adhesion of beneficial bacteria to plant roots for enhanced crop growth and stress management

Authors: Martin Palmqvist, Sarosh Bejai, Johan Meijer, Gulaim Seisenbaeva, Vadim Kessler

Supplementary figure 1

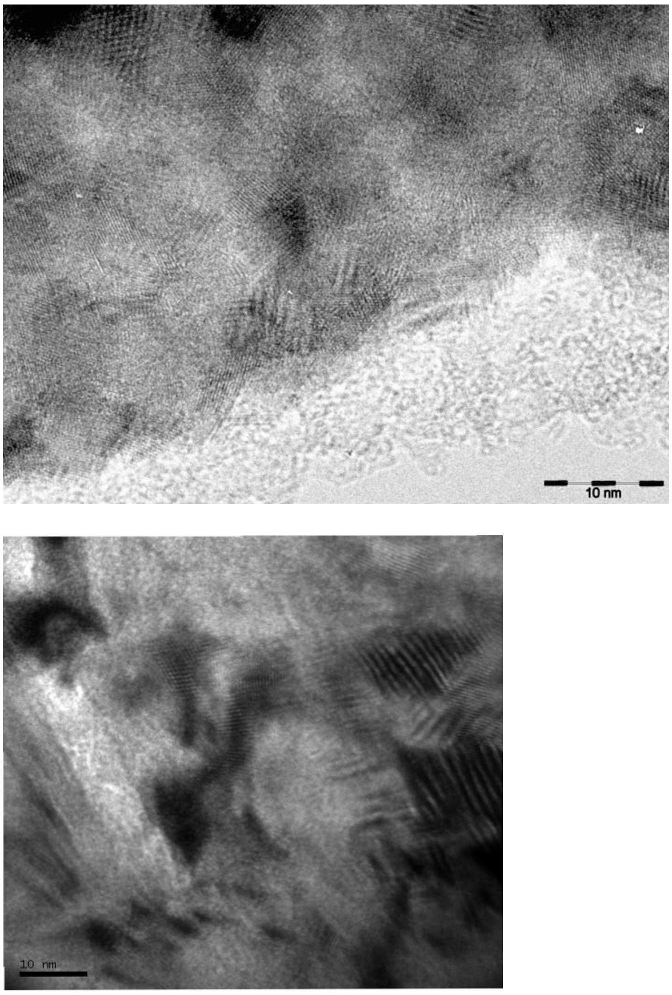

Figure S1. TEM images of nanoparticles. Above: Captigel nanoparticles with fringes showing atomic layers from the anatase crystal nucleus with an amorphous shell. Below: TiBALDH synthesised nanoparticles.

Supplementary figure S2

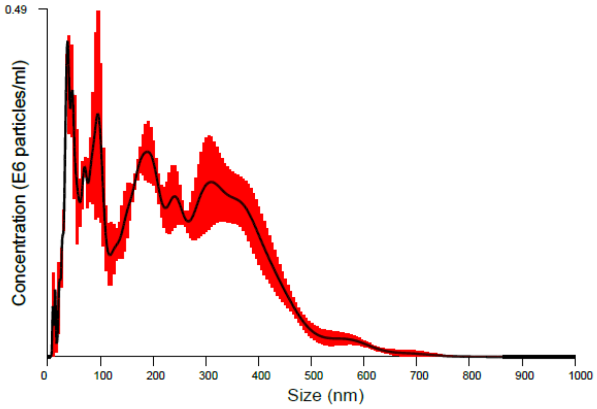

Figure S2. Nano tracking analysis, of TiBALDH synthesized nanoparticles in M9-minimal salts medium, with Nanosight 300.

Supplementary figure S3

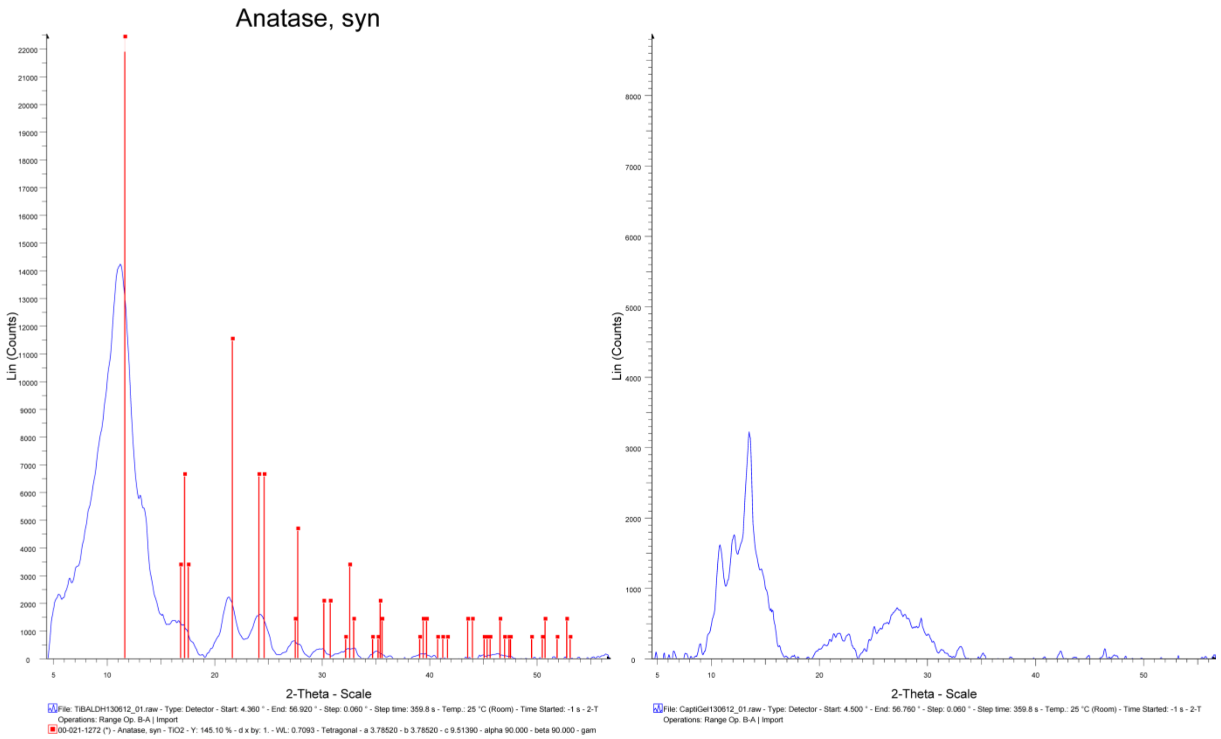

Figure S3. XRD results. Left are TiBALDH synthesized particles. Right is showing the amorphous Captigel nanoparticles.

Supplementary figure S4

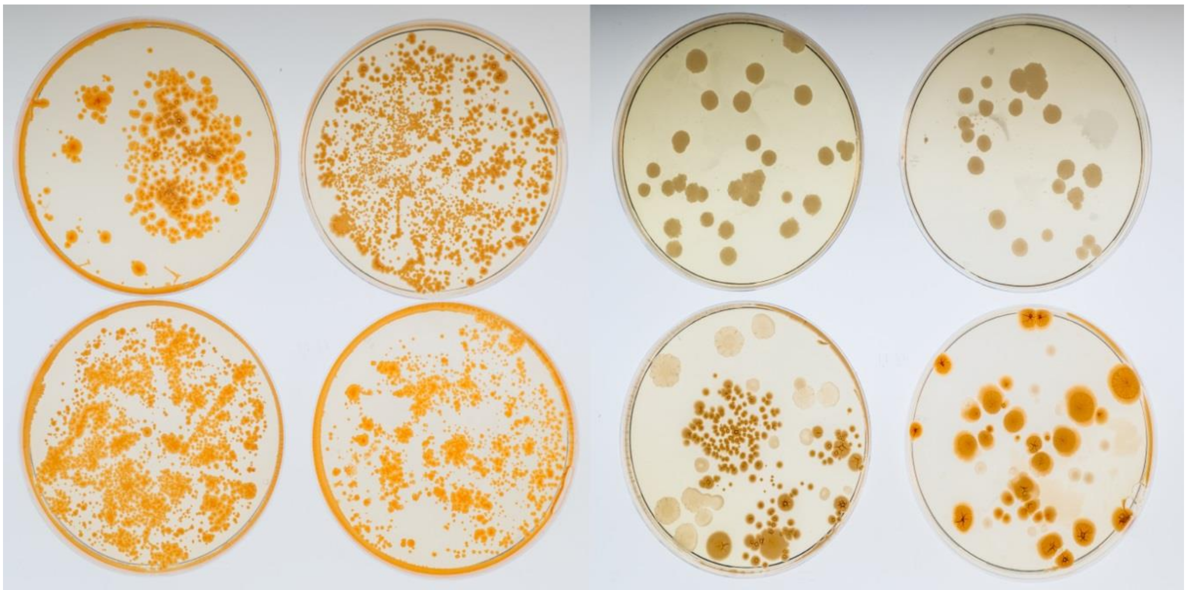

Figure S4. The four plates on the left represent control samples and the four plates on the right show bacteria mired together with TNs. The SEM-image in the supplementary figure 5 was taken from one of the orange spots in the bottom right plate. The spots that were not orange could physically not be opened up to show the presence of bacteria inside the even surface shell.

Supplementary figure S5

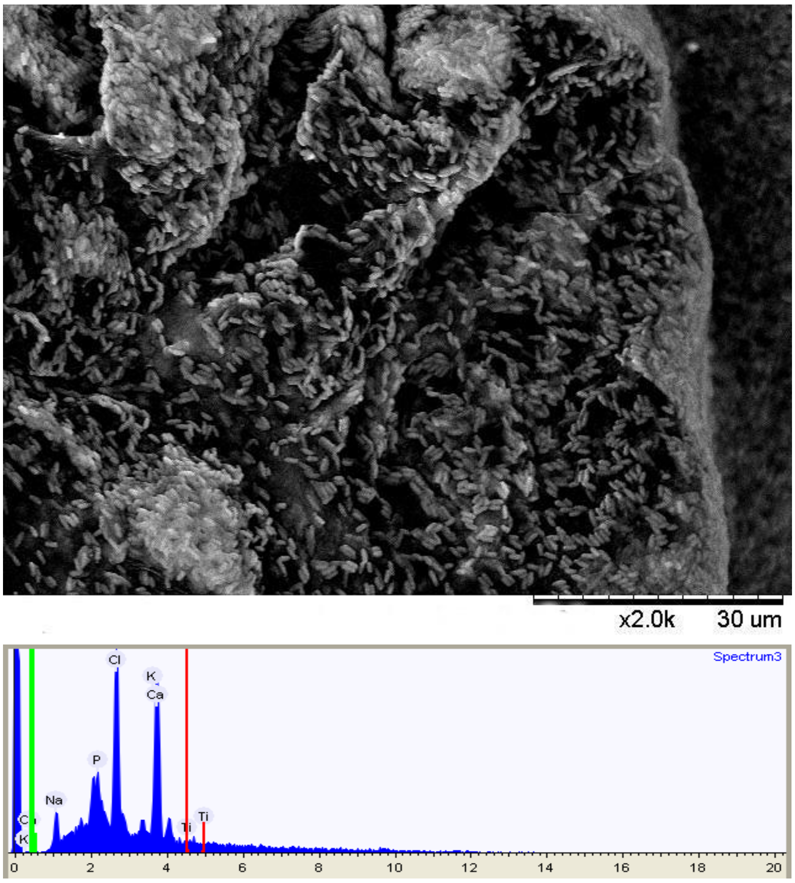

Figure S5. Above: SEM-image of bacteria in cluster with TNs after growth in plate reader for 16 hours and later on LB-agar for 48 hours, magnified 2000 times. Under: The EDS-analysis spectrum of bacteria in cluster with TNs after growth in plate reader for 16 hours and later on LB-agar for 48 hours. Titanium constituted 0.6 weight percentage of detected elements.

Supplementary figure S6

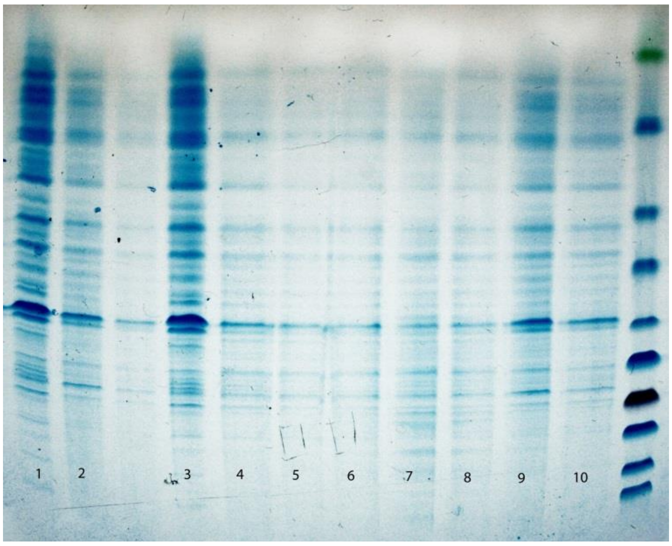

Figure S6. SDS-Page gel protein separation of bacteria cultures after plate reader. **1**. LB-TiBALDH 50 µg ml^{-1}. **2**. LB-TiBALDH 10 µg ml^{-1}. **3**. LB only. **4**. M9 only. **5**. M9 with ammonium lactate equivalent to 50 µg ml^{-1}. **6**. M9 with ammonium lactate equivalent to 10 µg ml^{-1}**.** **7**. M9 with sodium lactate equivalent to 50 µg ml^{-1}. **8**. M9 with sodium lactate equivalent to 10 µg ml^{-1}. **9**. M9 with TiBALDH 50 µg ml^{-1}. **10**. M9 with TiBALDH 10 µg ml^{-1}.

Supplementary figure S7

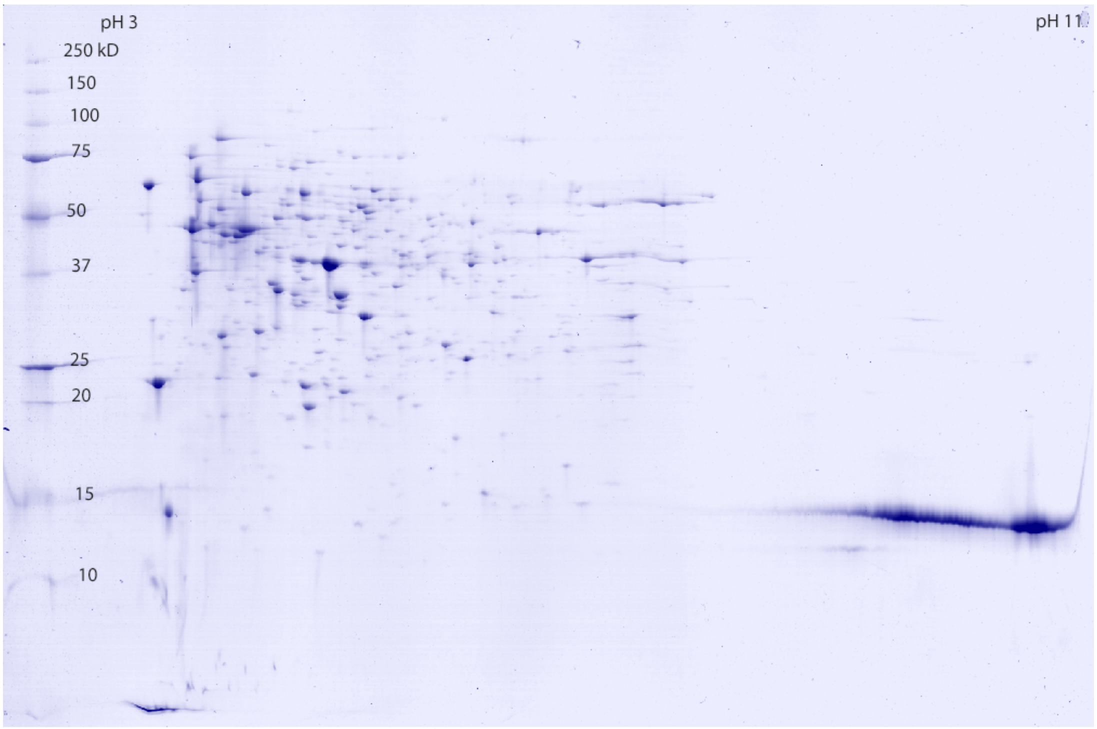

130826_JohanMeijer_M9

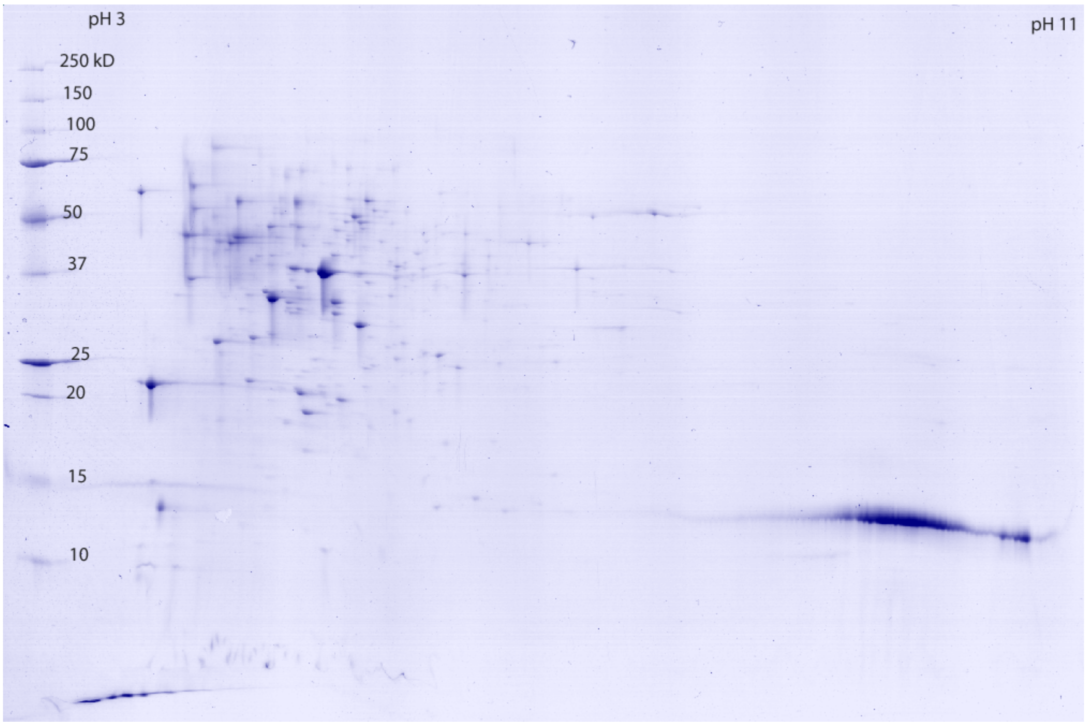

130826_JohanMeijer_tiB 50

Figure S7. Comparison of *Bacillus* proteome derived from cells grown in M9 medium without (top) or with titania nanoparticles (bottom) using two dimensional gel electrophoresis.

Supplementary figure S8

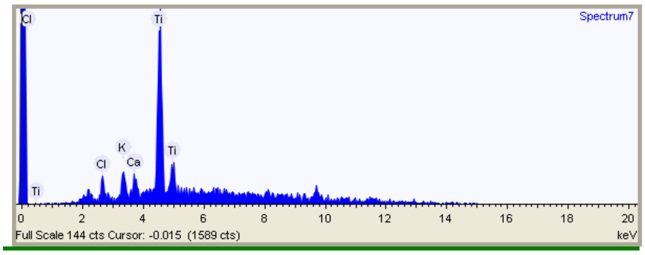

Element	Weight %
Chlorine	4.0
Potassium	6.3
Calcium	4.5
Titanium	85.2

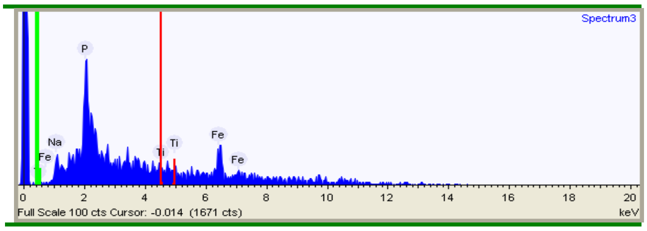

Element	Weight %
Sodium	18.6
Phosphorus	31.3
Titanium	3.0
Iron	47.2

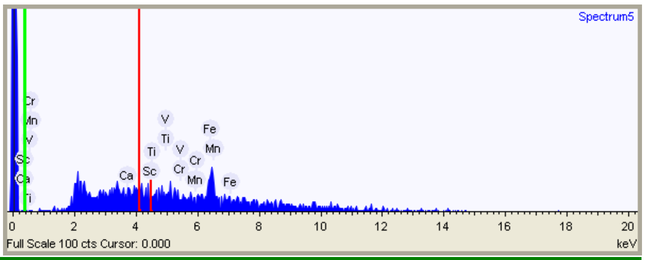

Element	Weight %
Calcium	7.5
Scandium	2.4
Titanium	0.0
Vanadium	2.6
Iron	87.6

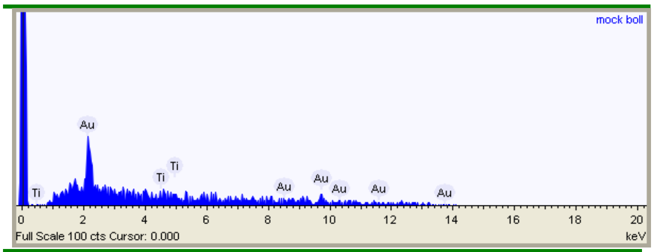

Element	Weight %
Titanium	0.0
Gold	100.0

Figure S8. Energy dispersive X-ray spectroscopy from SEM. From top to bottom: Captigel image in the article; TiBALDH image in the article; control with bacteria only and negative control.

Supplementary figure S9

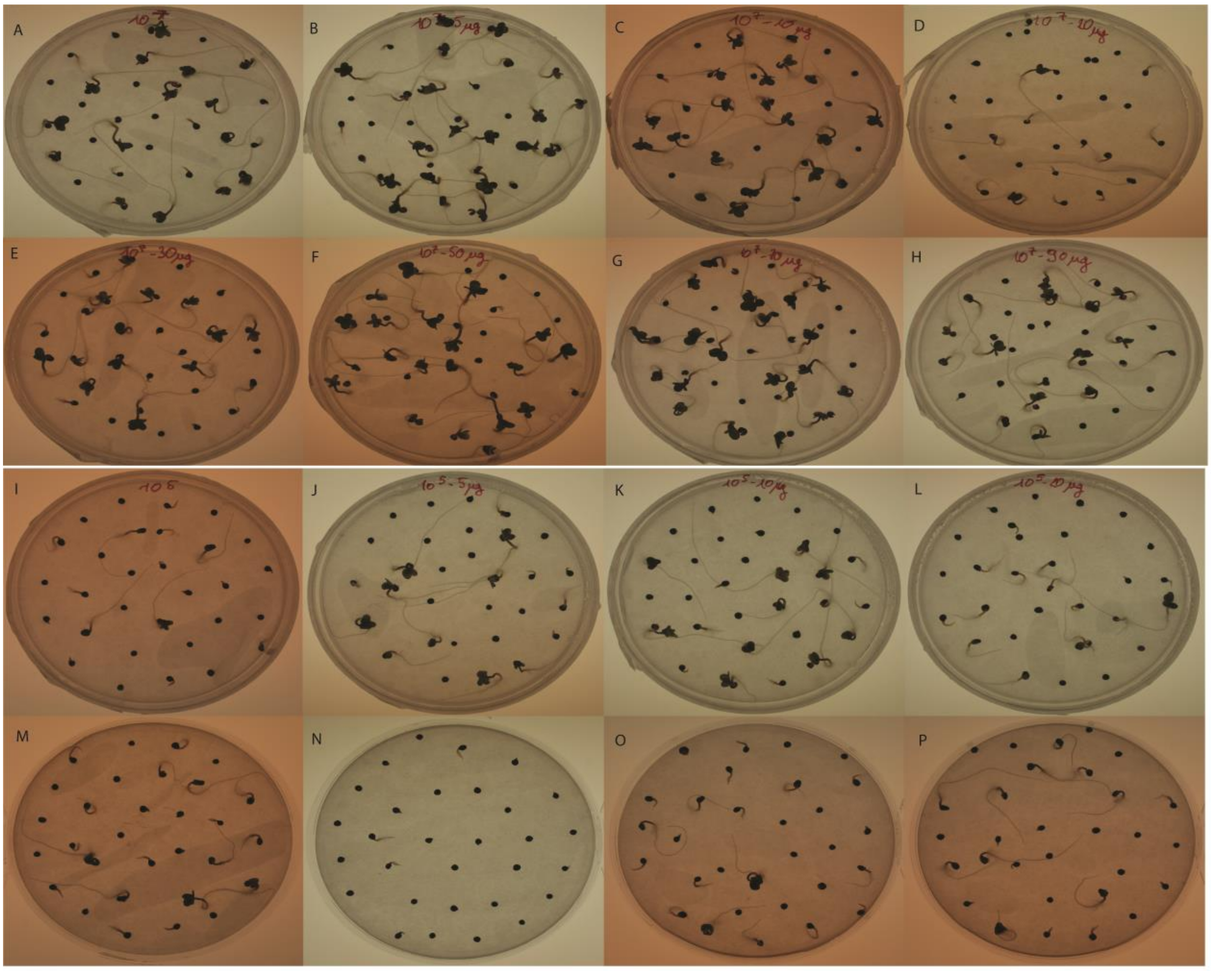

Figure S9. Rape seeds germination. A-H with 10^7 CFU ml^{-1}. I-P with 10^5 CFUs ml^{-1}. Both with the increasing concentration series of TNs: 0, 5, 10, 20, 30, 50, 70 and 90 µg/ml.

Supplementary table ST1. Means (n=12) ± standard deviation of quantification of the bacteria on roots with or without TNs with CFU counts on LB-plates or as estimated with SEM. Showing: Absolute CFU-count in mean per plate, CFU per mg root dry weight, images per replicate which contain bacteria and images per replicate that contains a colony of bacteria.

Treatment	CFU	CFU/mg	$SEM_{bacteria}$	SEM_{colony}
**TNs**	236±182	0.618±0.3	15.6±3.3	1.72 ±1.7
**Control**	198±91	0.7397±0.4	13.7±2.2	0.33±0.6
**P-value**	0.525	0.3994	0.129	0.018

Supplementary table ST2. List of target genes and primers used for qPCR.

Sequence accession number	Primer sequence	Amplicon length	Gene	Organism
NC_003074.8	CGATGGATCTGGAAAGGTTC<br>AGCTCCACAGGTTGCGTTAG	61 BP	Ubiquitin 5	Brassica Napus
U03393.1	ATCACTGCCGGTGGTTACTC<br>CGACACCCTTGATTTGGTCT	109 BP	Cutab1	Alternaria
NC_022081	GATCAGCTGGGGATCAAAAA<br>GGGCCAGAATGAGTACAGGA	94 BP	tetB	Bacillus Amyloliquefaciens 5113

Supplementary video S1

Bacterial colony treated with TiBALDH.wmv

Video S1. A compilation of images from SEM on the bacteria mired together with titania nanoparticles.
